# Admission pattern, treatment outcomes, and associated factors for children admitted to pediatric intensive care unit of Tikur Anbessa specialized hospital, 2021: a retrospective cross-sectional study

**DOI:** 10.1186/s12871-021-01556-7

**Published:** 2022-01-06

**Authors:** Ashenafi Seifu, Oliyad Eshetu, Dawit Tafesse, Seyoum Hailu

**Affiliations:** 1 Department of Anesthesiology, College of Health Sciences, Addis Ababa University, Addis Ababa, Ethiopia; 2 Department of Anesthesiology, College of Health and Medical Sciences, Hawassa University, Hawassa, Ethiopia; 3 Department of Anesthesiology, College of Health Sciences, Dilla University, Dilla, Ethiopia

**Keywords:** Pediatric intensive care unit, Admission pattern, Treatment outcome, Associated factor

## Abstract

**Background:**

Assessement of the pattern of admission and treatment outcomes of critically ill pediatrics admitted to pediatric intensive care units (PICU) in developing countries is crucial. In these countries with resource limitations, it may help to identify priorities for resource mobilization that may improve patient service quality. The PICU mortality rate varies globally, depending on the facilities of the intensive care unit, availability of experties, and admission patterns. This study assessed the admission pattern, treatment outcomes, and associated factors for children admitted to the PICU.

**Methods:**

A retrospective cross-sectional study was implemented on 406 randomly selected pediatrics patients admitted to the PICU of Tikur Anbessa Specialized Hospital from 1-Oct-2018 to 30-Sept-2020. The data were collected with a pretested questionnaire. A normality curve was used to check for data the distribution. Both bivariable and multivariable analyses were used to see association of variables. A variable with a *p*-value of < 0.2 in the bivariable model was a candidate for multivariate analysis. The strength of association was shown by an adjusted odds ratio (AOR) with a 95% Confidence interval (CI), and a *p*-value of < 0.05 was considered statistically significant. Frequency, percentage,and tables were used to present the data.

**Results:**

A total of 361 (89% response rate) patient charts were studied, 197 (54.6%) were male, and 164(45.4%) were female. The most common pattern for admission was a septic shock (27.14%), whereas the least common pattern was Asthma 9(2.50%). The mortality rate at the pediatric intensive care unit was 43.8%. Moreover, mechanical ventilation need (AOR = 11.2, 95%CI (4.3–28.9), *P* < 0.001), need for inotropic agents (AOR = 10.7, 95%CI (4.1–27.8), *P* < 0.001), comorbidity (AOR =8.4, 95%CI (3.5–20.5), P < 0.001), length of PICU stay from 2 to 7 days (AOR = 7.3, 95%CI (1.7–30.6), *P* = 0.007) and severe GCS (< 8) (AOR = 10.5, 95%CI (3.8–29.1), *P* < 0.001) were independent clinical outcome predictors (mortality).

**Conclusion:**

The mortality rate at the PICU was 43.8%. Septic shock, and meningitis were the common cause of death and the largest death has happened in less than 7 days of admission.

## Introduction

A pediatric intensive care unit (PICU) is a unit in a hospital where most critical children with life-threatening conditions receive pediatric care [1]. Admission to intensive care unit (ICU) may be required if the patient experiences hemodynamic instability requiring frequent monitoring of vital signs, invasive hemodynamic monitoring, rapid titration of intravenous medication with concurrent monitoring, and respiratory support in the ICU. This may significantly improve the quality of care and outcomes of critically ill and injured patients, predominantly in high-resource settings [2]. However, critical care practice is very difficult in developing nations where health needs frequently exceed available resources, and most critical health care institutions are still in their early stages of development [3].

Patterns of admission and treatment outcomes in the PICU vary in different regions of the globe. The most common admission reason for pediatric cases to the PICU are trauma, postoperative treatment, complicated meningitis, cardiovascular, neurological, acute respiratory distress syndrome (ARDS), and septic shock [1, 4–7].

The ICU outcomes were determined by clinical condition at admission, patient age, comorbidity, level of pre-hospital and emergency trauma care, and factors reported during ICU admission, such as the use of mechanical ventilation, level of consciousness, duration of ICU stay, as well as complications during ICU stay, such as circulatory and ventilator-related respiratory complications [7–10].

The shortage of basic health care facilities, mainly in African countries, causes a distinctly different continuum of clinical problems. In different regions of Africa, the pattern of ICU admission varied far too widely from country to country or region to region. In sub-Saharan Africa, mortality rates of critically ill patients treated in resource-limited ICU are often high because of the shortage of skilled personnel, equipment, and supply materials [11]. The WHO report shows that the major causes of death among children under the age of five in developing countries are preventable and curable diseases if treatment is optimized [12].

The prevalence of PICU mortality also varied in different regions of the globe. In Brazil, India, and Nigeria the prevalence of PICU mortality was 10.3%, 2.1, and 34.6% respectively, while in Gondar (Northwest Ethiopia), Ayder referral hospital (North Ethiopia), and Jimma (Southwest Ethiopia), PICU mortality rate were 30.9, 8, and 40% respectively [1, 5–8, 13].

The major purpose of the pediatric intensive care unit is to reduce death by closely monitoring and treating severely ill children who are considered to be at high risk of death. The effectiveness of therapy will be determined by evaluating the outcomes of medical treatments. The mortality determinants have varied across the globe and even this may was serious in our study.

There is a limitation of data on admission patterns, treatment outcomes, and associated factors for pediatric patients admitted to the PICU in our country. The findings of this study will help to develop prevention and treatment strategies for the major preventable causes of deaths among pediatric patients admitted to the PICU. Therefore, the primary objective of this study was to assess the pattern of admission, and treatment outcomes of pediatric patients admitted to the PICU and the secondary objective was assessing associated factors for the treatment outcomes.

## Methods and materials

### Study area

This research was carried out at Tikur Anbessa Specialized Hospital (TASH) in Addis Ababa, the capital city of Ethiopia. TASH is Ethiopia’s largest referral hospital in the country. It has 4 PICU tables, and 13 adult ICU beds, 28 fully functional NICU beds and 13 adult ICU beds as multipurpose for both medical and surgical cases. The study was carried out after ethical approval was obtained from Addis Ababa University College of Health Sciences. The methodology in this study followed the International Committee of Medical Journal Editing (ICMJE) Statement that follows the recommendations for the conduct, reporting, editing, and publication of scholarly work in the medical journal. A research registry at WWW.researchregistry.com registered this study with UIN: research registry 7081.

### Study design and period

An institutional retrospective cross-sectional study was conducted on the data available from 1-Oct- 2018 to 30-Sept- 2020.

### Source and study population

All pediatric patients that admitted into the pediatric intensive care unit (PICU) of Tikur Anbessa specialized Hospital were our source population, while pediatrics patients admitted to the PICU during the study period that fulfilled inclusion criteria were our study population.

### Study variables

#### Dependent variable

Treatment Outcomes at PICU discharge (survived or died).

#### Independent variables

Age, Gender, Clinical diagnosis at admission, Presence of comorbid illness, Source of admission, frequency of admission, Category of admission, Mental status, Intervention during ICU stay, and length of ICU stay.

### Eligibility criteria

All pediatric age group patients who were admitted to the PICU of Tikur Anbessa Specialized Hospital during the study period were included, while those who died on arrival or died within two hours of admission were excluded.

### Sample size and sampling technique

The sample size was calculated using the single population proportion formula, and a 5% margin of error at a 95% confidence interval. We calculated sample size for both outcome variables and we get the largest sample size with pediatrics intensive care unit mortality which was 40% [5].

$$\mathbf{n}=\frac{\mathbf{z2}\boldsymbol{\upalpha } /\mathbf{2}\mathbf{p}\left(\mathbf{1}-\mathbf{p}\right)}{{\boldsymbol{w}}^{\mathbf{2}}}$$

Where n = sample size, *p* = 0.4, q = 1-*p* = 0.6, w = 0.05 and Z α/2 = 1.96.

Substituting the values in the equation yields a sample size of 369, plus 10% attrition rate (369 + 37 = 406) we got a total sample size of 406. From situational analysis, 780 pediatrics were admitted to the pediatric intensive care unit of the Hospital from 1-Oct-2018 to 30-Sept-2020.

After selecting the first study participant by lottery method, a systematic random sampling technique was used to select study participants with k^th^ skip interval, where K=N/n. = 780/406 = 2. We selected every third patient chart from the data set until we got the required sample of 406.

N- Total study population admitted to the PICU from 1-Oct-2018 to 30-Sept- 2020.

n- Total calculated sample size.

### Data collection protocols and procedures

The ethical clearance and permission for data collection was granted before starting data collection. The data were collected by three bachelor nurses and one master’s holder who supervised the data collection process. Training on data collection was given for data collectors before starting the data collection. To ensure content validity, the questionnaire was adapted from previous related studies, tested for reliability, and experts’ suggestions were included. The medical records of the patients were examined as per the objectives of the study. Finally, the patient’s charts were appropriately replaced in their original location after data collection.

### Operational definition

#### Clinical outcome

Indicate either patient survived or died at the time of discharge.

#### Survived

Patients who survived during ICU stay, including patients who were improved, transferred to the pediatric ward or discharged.

#### Non-survived

Patients who are not alive at the time of discharge.

#### Length of ICU stays (LOS)

Was a period in hours, days, or months the patients stayed in ICU from admission time to discharge time.

ICU Intervention: prevention of vital organ function failure and treatment of critically ill patients and applied in an appropriately staffed and equipped ICU setting.

ICU Mortality: was calculated as the number of deaths of patients given particular diagnoses divided by the total number of patients with that diagnosis.

### Data quality assurance

Before the actual data collection, the questionnaire adapted from previous studies was translated to Amharic (one of the local official languages) by a language expert, then this Amharic version was translated back to English by another language expert to increase its releability. A pretest was done on 5% of the sample size (21 participants) in Minilik II referral hosptal before actual data collection. Investigators supervised data collection processes. Collected data were checked for completeness, accuracy, and clarity. Complete and coded data were entered into Epi Info version 7.0. Data clean-up and cross-checking were done before analysis on SPSS version 26.

### Data processing and analysis

Data were checked manually for completeness, coded and entered into Epi info version 7 computer program. It was then exported to SPSS version 26.0 for analysis. The data distribution was checked with a normality curve. Bivariable and multivariable logistic regression models were carried out to examine the effect of explanatory variables over the outcome variable. The variables with a *p*-value of less than 0.2 on bivariable model were taken into multivariable analysis. In multivariable analysis p-value of less than 0.05 was used as a cut off point for the presence of association. Strength of association was measured by an Adjusted Odds ratio with a 95% confidence interval. Frequency, percentage, tables, and figures were used to present the data.

## Results

### Socio-demographic and clinical characteristics of admitted pediatrics

A total of 406 patient charts were available for data collection, and 361 had full information required, this makes it 89% response rate, which is acceptable since we added a 10% attrition rate. From the total patients, 197 (54.6%) were males and 164 (45.4%) were females. Pediatric ward took the highest source of PICU admission 131(36.3%) followed by emergency pediatric unit 105(29.1%), operation room 90(24.9%), surgical ward 18(5%), and recovery room 17(4.7%). Three hundred and eithen (88.1%) were admitted for the first time, while 43(11.9%) of pediatric patients were re-admitted to the PICU. The majority of them stayed in PICU for 1–7 days. A total of 158 (43.8%); 84 (23.27%) males, and 74 (20.23%) females were died. The most common reason for immediate death in PICU was multi-organ failure 86(23.82%) followed by cardiac arrest 67(18.56%) and respiratory failure 5(1.38%) (Table 1**).**

**Table 1:** Socio-demographic and clinical outcomes of pediatric patients admitted to the PICU during the study period (*n* = 361)

Variable	Admission	Survive	Death
**Age:**	N(%)	N(%)	N(%)
**Birth-1 Month**	77(21.34)	35(9.7)	42(11.64)
**1 Month-2 year**	118(32.68)	73(20.2)	45(12.48)
**2 Year-12 years**	166(45.98)	95(26.3)	71(19.68)
**Total**	361(100)	203(56.2)	158(43.8)
**Gender:**	N(%)	N(%)	N(%)
**Males**	197(54.6)	113(31.31)	84(23.27)
**Females**	164(45.4)	90(24.93)	74(20.50)
**Total**	361(100)	203(56.2)	158(43.8)
**Length of ICU stay**	N(%)	N(%)	N(%)
**< 2 days**	47(13.02)	27(7.48)	20(5.54)
**2–7 days**	128(35.46)	64(17.72)	64(17.72)
**7–14 days**	101(27.98)	58(16.06)	43(11.91)
**14-28 days**	38(10.52)	19(5.26)	19(5.26)
**> 28 days**	47(13.02)	35(9.70)	12(3.32)
**Total**	100%	203(56.2%)	158(43.8)

Foot note- N = frequency, % = Percentage

### Diagnosis at admission to pediatric intensive care unit

The current study found that, the three most common reason to the PICU admission were septic shock 98(27.14%), meningitis 67(18.56%), and congestive heart failure (CHF) 44(12.19%). The other causes of PICU admission were post-operative care 34(9.42%), severe pneumonia 22(6.09%), upper airway obstruction 19(5.26%), traumatic brain injury (TBI) 18(4.98%), acute respiratory distress syndrome (ARDS) 17 (4.71%), cardiogenic shock 13(3.60%), acute glomerular necrosis 10(3.60%), Gillie barre syndrome (GBS) 10(2.77%), and Asthma 9(2.50%) (Table 2**).**

**Table 2:** Top five causes of admission and their outcomes among pediatric admitted to the PICU during the study period(*n* = 361)

Causes of Admission	Admission (%)	Death (%)	Case fatality
Septic shock	98(27.15)	63(17.45)	64.29%
Meningitis	67(18.56)	41(11.36)	61.19%
Cardiovascular disease	44(12.19)	24(6.65)	54.54%
Post-operative	34(9.42)	8(2.22)	23.52%
Severe pneumonia	22(6.09)	1(0.27)	4.55%

### Clinical outcome on discharge from pediatric intensive care unit

The clinical outcomes of the PICU patients was evaluated in this study. Accordingly, among pediatric patients admitted to the PICU 183 (50.69%) were improved, 158 (43.80%) were died, 13 (3.60%) left against medical advice, and 7 (1.94%) were referred to other hospitals.

### Associated factors with mortality in the pediatric intensive care unit

The possible associated factors for such high mortality rate in the PICU were studied. These factors include; level of consciousness at amdssion, admission diagnosis (Septic shock, Meningitis, Cardiovascular disease, severe pneumonia and Acute Respiratory Distress Syndrome /ARDS/), ICU stay duration, presence of co-morbid illness, the need for mechanical ventilators, inotropic support, and blood transfusion were associated with mortality in bivariable analysis, and these factors were exported to multivariable analysis for a possible co-founders.

The findings of a multivariable analysis showed that, severe level of consciousness (GCS < 8) (AOR = 10.52, 95%%CI (3.80–29.05), *P* < 0.001) and moderate level of consciousness (GCS 9–12) (AOR = 4.19, 95%CI (1.47–11.99), *P* = 0.007) had about 11 times and 4 times odds of mortality rate than those with mild level of consciousness (GCS 13–15) respectively.

Patients’ stay in the PICU for 2–7 days (AOR = 7.3, 95%CI (1.73–30.55), *p* = 0.007), for 14–28 days (AOR = 7.02 95%CI (1.08–45.19), *P* = 0.04), < 2 days(AOR = 5.81, 95%CI (1.07–31.34), *P* = 0.041), and 7–14 days (AOR = 5.42, 95%CI (1.18–24.80), *P* = 0.029) had about 7 times, 7 times, 6 times, and 5 times odds of death than those who stayed in the PICU for > 28 days respectively.

Presence of other coexisting diseases during admission (AOR = 8.38, 95%CI (3.42–20.5), *P* < 0.001) had 8 times odds of death than those without coexisting diseases at admission into the PICU.

Patients who needed for mechanical ventilation (AOR = 11.08, 95%CI (4.25–28.87), *p* < 0.001), and those who needed for inotropic support (AOR = 10.67, 95%CI (4.09–27.81), *P* < 0.001) had equally about 11 times odd of death than those who did not needed either mechanical ventilation or inotropic support (Tables 3 and 4**)**.

**Table 3:** Factors associated with pediatric patients mortality at the PICU during the study period (*n* = 361)

Variable	Outcome				
	Survived (%)	Dead (%)	COR	AOR (95%CI)	P-value
**Level of GCS**					
12–15	144(39.88)	33(9.14)	1	1	
9–12	45(12.47)	35(9.70)	3.39(1.89–6.07)	4.185(1.47–11.99)	0.007^a^
< 8	14(3.89)	90(24.93)	28(14.23–55.28	10.51(3.81–29.05)	< 0.001^a^
**Admission diagnosis**					
Septic shock	35(9.69)	63(17.45)	5.85(2.39–14.30)	51.05(0.22–.93)	0.942
Meningitis	26(7.20)	41(11.36)	5.12(2.01–13.02)	1.61(0.32–7.97)	0.55
CVD	20(5.54)	24(6.65)	3.90(1.44–10.49)	2.38(0.07–2.59)	0.35
Severe Pneumonia	21(5.82)	1(0.27)	6.45(0.02—1.34)	30(0.001–1.81)	0.095
ARDS	10(2.77)	7(1.94)	2.27(0.65–7.93)	1.9(0.78–3.66)	0.52
UAO	19(5.26)	0(0)			
TBI	4(3.87)	4(1.11)	1.08(0.23–3.63)		
Cardiogenic shock	10(2.77)	3(0.83)	1.02(0.215–4.43)		
AGN	7(1.93)	3(0.83)	1.39(0.29–6.67)		
GBS	9(2.50)	1(0.27)	2.77(0.24–3.30)		
Asthma	6(1.66)	3(0.83)	1.62(0.33–8.02)		
Post-operative	26(7.20)	8(2.22)	1		

^a^ Statistically significant

**Table 4:** Factors associated with pediatric patients mortality at the PICU during the study period (n = 361)

Variable	Outcome				
	Survived (%)	Dead (%)	COR	AOR (95%CI)	P-value
**ICU stay duration**					
Less than 2 days	27(7.48)	20(5.54)	2.16(0.90–5.17)	5.81(1.07–31.34)	0.041^a^
2–7 days	64(17.72)	64(17.72)	2.91(1.38–5.18)	7.27(1.73–30.55)	0.007^a^
7–14 days	58(16.06)	43(11.91)	2.16(1–4.64)	5.42(1.18–24.80)	0.029^a^
14–28 days	19(5.26)	19(5.26)	2.91(1.17–7.27)	7.02(1.08–45.19)	0.04^a^
> 28 days	35(5.70)	12(3.32)	1	1	
**Comorbidity**					
No	153(42.38)	31(8.59)	1	1	
Yes	50(13.85)	127(35.18)	12.53(7.55–20.79)	8.38(3.42–20.5)	< 0.001^a^
**Need for MV**					
No	136(37.67)	16(4.43)	1	1	
Yes	67(18.56)	142(39.34)	18.02(9.95–32.63)	11.08(4.25–28.87)	< 0.001^a^
**Inotropic need**					
No	177(49.03)	59(16.34)	1	1	
Yes	26(7.20)	99(27.43)	11.42(6.72–19.26)	10.67(4.09–27.81)	< 0.001^a^
**Transfusion need**					
No	181(50.14)	132(36.56)	1	1	
Yes	22(6.09)	26(7.20)	1.62(0.88–2.98)	1.24(0.39–3.93)	0.71
Vasopressor need					
No	175(48.48)	80(22.16)	1		
Yes	28(7.75)	78(21.61)	6.1(3.67–10.1)		

Foot note: MV = mechanical ventilation. ^a^ = Statistically significant, 1 = reference

## Discussion, and conclusions

### Discussion

This study evaluated the pattern of admission into the PICU,treatment outcomes, and associated factors. A total of 406 patient charts were available for data collection, and 361 had full information required, this makes it 89% response rate, which is acceptable since we added a 10% attrition rate. From the total patients, 197 (54.6%) were males and 164 (45.4%) were females.

In this study, the maority of pediatrics admitted were aged between 2-year and 5-years. This is inline with study conducted in India which stated that more than 36% patients were between 1 and 5 year, and about 32% were infants [14]. Similar study conducted in Nigeria showed different from our study. They found that more than 50.7% of patients admitted into pediatric intensive care were infants [7]. Despite such discripancies, our study as well as previous studies did not found statistically significant mortality rate associated with age. The vast majority of our study participants were children aged between 2-year, and 5-years.

The majority of patients admitted into our PICU stayed for 1–7 days. The most common reason for immediate death in PICU was multi-organ failure 86(23.82%) followed by cardiac arrest 67(18.56%) and respiratory failure 5(1.38%).

The determinants of the PICU outcome were based on the seriousness of the condition at admission, patient age, presence of comorbidities, the quality of pre-hospital and emergency trauma care; factors reported during the PICU admission like the use of mechanical ventilation, level of consciousness, duration of the PICU stay; complications during the PICU stay like circulatory and ventilator-related respiratory complications [7–10]. It is challenging to give effective, and adequate care for the children admitted to the PICU without knowing the causes of admission. Hence, for the health system to be successful, it is important to know the key obstacles in the way to improve patient health and how this challenge can be overcome [15].

Sixty-one percent (61%) of pediatrics patients were admitted to the PICU with a medical diagnosis such as Septic shock 27.14%, meningitis 18.56%, and cardiovascular disease 12.9% or so in our study. This finding is in line with studies done in Greek, and Ayder referral hospitals where the most common reasons of admission were pathological diseases [6, 16]. This was also inline with a study conducted in Eritirea, which states that the vast majority of PICU admissions were due to medical problems (80.5%) and out of this infectious diseases accounted for 50% of the cases [17]. The possible causes of admission into the PICU in our study were different from a study done in general ICU of Jimma University specialized hospital (54.7%) where surgical, and trauma patients were the dominant reason for admission [5]. This might be due to the difference in the study population, we studied pediatrics and they studied the general intensive care unit or this difference may be attributed to the fact that our study was carried out in the biggest referal hospital in the country.

The present study found that the overall mortality rate in the PICU was 158 (43.8%). The rest 183(50.69%) were improved, 13 (3.60%) were left againist medical advice, and 7 (1.94%) were referred to other hospitals. The mortality rate was higher than similar studies, this high mortality rate in our study may be explained by resource scarcity, and our hospital is the biggest specialized referral hospital where critical patients are admitted from zones, regions, and even from other tertiary and specialized hospitals that may result in delayed admission, and poor clinical outcome [18].

The totatal of 361 patients cases in the PICU were studied. Of the total patients admitted to the PICU, 57.89% were ventilated mechanically (*p* < 0.001) which is higher than the study done in Nepal (30%), the general intensive care unit of Jimma university specialized hospital (37.1%), Ayder referral hospital (4%), and Gondar university hospital (10%) [1, 5, 6, 19]. The high proportion needed for Mechanical ventilation in our setup can be explained by the fact that our hospital is a specialized referral hospital where critical patients are referred from districts, zones, regions, and even from other tertiary, and specialized hospitals.

The mortality rate among patients on mechanical ventilation was 67.94% (*p* < 0.001) which is in line with research done in Nepal (68%) [19], however, it is somehow higher than a study done in Gondar (60.6%) [1]. The need for mechanical ventilation was an independent associated factor for mortality in the PICU (*p* < 0.001).

Patients admitted to the PICU usually have coexisting diseases other than primary causes of admission [20]. Coexisting diseases had a remarkable effect on acute illness, complications in the PICU, and clinical outcomes [21]. The overall mortality rate of patients with co-morbidity was 71.75% which was higher than a study done in the west of Scotland (24–53%) [20]. This might be due to resource scarcity and lack of early treatment for comorbidities in our health system.

Length of stay in the PICU for 2–7 days had a statistically significant association with mortality (*p* = 0.007) than those who stayed for greater than 28 days. This, however, opposes a study done in Ayder referral hospital which stated mortality was higher in patients who stay greater than 28 days [8]. This discrepancy might be attributed to the fact that most patients who stayed in the PICU for 2–7 days were on mechanical ventilation in our study, which we found it to be an statistically significant associated factor for the PICU mortality with *p* < 0.001.

We further found that, the need for inotropic support in PICU was significantly associated with high mortality rate than those who did not required the inotropic support. This is in line with studies done in different parts of the world [5, 6, 22].

The current study found that severe (GCS < 8) and moderate (GCS 9–12) levels of consciousness were significantly associated with the PICU mortality than mild (GCS 13–15) level of consciousness (*p* < 0.001) and (*p* = 0.007). This finding is similar to a study done in Ayder referral hospital (North Ethiopia) [6].

### Strength of the study

The calculated sample size for our study is adequate to draw generalizations.

### Limitations of the study

The possible limitation of our study is that it is retrospective and done in a single study setting. The study needs to be conducted in multi-center for the future.

## Conclusion

In conclusion, the mortality rate at the PICU was 43.8%. Septic shock and meningitis were the common causes of death, and the largest death has happened in less than 7 days of admission. The need for mechanical ventilation, for inotropes, presence of comorbidity, length of PICU stay from 2 to 7 days and lower GCS were significantly associated with mortality in the PICU. Our implication is that, early PICU, well equiping the PICU with both experts, and material resources, propmt management for primary presenting disease as well co-existing diseases are crucial to improove the PICU care.

## Recommendations

We like to recommend that, all governmental and non-governmental stakeholders should work on generating awareness in the community for early health care seeking (admission), and for strengthening the quality of the PICU services. The study setup has to increase the PICU capacity to give adequate PICU care for patients with life-threatening conditions. The emergency trauma care service should have to be very active for timely transport of patients from hospitals. The basic and advanced capacity building strategies for PICU staffs should be implemented. We like to recommend the researchers to come to consider a multi-centered prospective study to minimize possible biases.

## Data Availability

The datasets used and/or analysed during the current study are available from the corresponding author on reasonable request.

## Abbreviations

AGN: Acute Glomerulonephritis
ARDS: Acute Respiratory Distress Syndrome
AOR: Adjusted Odds Ratio
CVD: Cardiovascular Disease
COR: Crude Odds Ratio
GCS: Glasgow’s Coma Scale
GBS: Gullian Barre Syndrome
ICU: Intensive Care Unit
LOS: Length of stay
MV: Mechanical Ventilation
PICU: Pediatric Intensive Care Unit
SPSS: Statistical package for social science
TASH: Tikur Anbessa specialized Hospital
TBI: Traumatic Brain Injury
UAO: Upper Airway Obstruction
WHO: World Health Organization
